# Retinoic acid signalling in fibro/adipogenic progenitors robustly enhances muscle regeneration

**DOI:** 10.1016/j.ebiom.2020.103020

**Published:** 2020-09-24

**Authors:** Liang Zhao, Jun Seok Son, Bo Wang, Qiyu Tian, Yanting Chen, Xiangdong Liu, Jeanene M. de Avila, Mei-Jun Zhu, Min Du

**Affiliations:** a Nutrigenomics and Growth Biology Laboratory, Department of Animal Sciences, and School of Molecular Bioscience, Washington State University, Pullman, WA; b State key Laboratory of Animal Nutrition, College of Animal Science and Technology, China Agricultural University, Beijing, China, 100193; c School of Food Science, Washington State University, Pullman, WA

**Keywords:** Fibro/adipogenic progenitors, Retinoic acid signalling, Muscle regeneration, Fibrosis, Adipogenesis, Obesity

## Abstract

**Background:**

During muscle regeneration, excessive formation of adipogenic and fibrogenic tissues, from their respective fibro/adipogenic progenitors (FAPs), impairs functional recovery. Intrinsic mechanisms controlling the proliferation and differentiation of FAPs remain largely unexplored.

**Methods:**

Here, we investigated the role of retinoic acid (RA) signalling in regulating FAPs and the subsequent effects on muscle restoration from a cardiotoxin-induced injury. Blockage of retinoic acid receptor (RAR) signalling was achieved through dominant negative retinoic acid receptor α (RARα403) expression specific in PDGFRα+ FAPs *in vivo* and by BMS493 treatment *in vitro*. Effects of RAR-signalling on FAP cellularity and muscle regeneration were also investigated in a high-fat diet-induced obese mice model.

**Findings:**

Supplementation of RA increased the proliferation of FAPs during the early stages of regeneration while suppressing FAP differentiation and promoting apoptosis during the remodelling stage. Loss of RAR-signalling caused ectopic adipogenic differentiation of FAPs and impaired muscle regeneration. Furthermore, obesity disrupted the cellular transition of FAPs and attenuated muscle regeneration. Supplementation of RA to obese mice not only rescued impaired muscle fibre regeneration, but also inhibited infiltration of fat and fibrotic tissues during muscle repair. These beneficial effects were abolished after blocking RAR-signalling in FAPs of obese mice.

**Interpretation:**

These data suggest that RAR-signalling in FAPs is a critical therapeutic target for suppressing differentiation of FAPs and facilitating the regeneration of muscle and other tissues.

**Funding:**

This study was supported by grants from the National Institutes of Health (R01-HD067449 and R21-AG049976) to M.D.

**Research in context**

**Evidence before this study**

Intramuscular fibro/adipogenic progenitors (FAPs) are generally quiescent but undergo rapid activation under pathophysiological conditions such as muscle injury in order to facilitate muscle regeneration. However, failure of apoptosis at the late stage of regeneration leads to fibrotic or adipogenic infiltration. Intrinsic mechanisms regulating the cellular states of FAPs remain largely undefined.

**Added value of this study**

Our findings delineate the importance of retinoic acid (RA) signalling in regulating cellular states of FAPs and muscle regeneration after injury. Blockage of RAR-signalling in FAPs leads to ectopic infiltration of adipocytes and impaired muscle fibre regeneration. RA treatment promotes cellular proliferation, but inhibits the adipogenic and fibrogenic differentiation of FAPs. Furthermore, RA-signalling in the skeletal muscle of obese mice is down-regulated while dynamic changes of FAPs during muscle regeneration are also impaired. Supplementation of RA to obese mice not only rescues impaired muscle fibre regeneration but also inhibits fatty and fibrotic infiltration through promoting the proliferation of FAPs at the early stage and increasing their apoptosis at the remodelling stage.

**Implications of all the available evidence**

RA-signalling mediators in FAPs represent novel therapeutic targets to inhibit the pathological infiltration of fatty and fibrotic tissues of skeletal muscle. Such complications are associated with various conditions including, but not limit to, muscular dystrophy, sarcopenia, and muscle degeneration due to obesity and diabetic conditions. In addition, because adipogenesis and fibrosis are not limited to muscle tissue, our data have wide clinical applications.

Alt-text: Unlabelled box

## Introduction

As the largest organ, skeletal muscle exerts multiple functions in organisms, including motility support, and the regulation of basal energy metabolism [1]. Fatty infiltration and fibrosis of skeletal muscle due to degenerative diseases, sarcopenia or incomplete regeneration from injuries progressively impair its physical and metabolic functions [2]. In an attempt to better understand preventative methods of such conditions, intensive research has been done on myogenic and non-myogenic progenitors involved in pathological muscle degeneration and regeneration.

As a tissue with high plasticity, adult skeletal muscle maintains an impressive capacity to regenerate after injury. Muscle regeneration is a well-coordinated process which involves multiple types of cells [3]. Satellite cells (SCs) have garnered most attentions because they are directly responsible for repair of damaged myofibers and the formation of new myofibers [4,5]. Myogenic functions of SCs are regulated by a group of non-myogenic cells in the local milieu including immune cells, vascular cells, and mesenchymal stem cells [3,6,7]. Paracrine signals originating from these non-myogenic cells orchestrate expansion and differentiation of SCs, and hence the progress of muscle repair. amongst these supportive non-myogenic cells, mesenchymal fibro/adipogenic progenitors (FAPs) reside in the interstitial space between myofibers and have been recognized as critical mediators for myogenic differentiation of SCs [8], [9], [10], [11].

FAPs are a group of progenitor cells expressing cell surface markers, stem cell antigen-1 (Sca-1) and platelet-derived growth factor receptor alpha (PDGFRα) [8,9,12]. While FAPs are largely quiescent under physiological conditions, acute injury rapidly stimulates their massive expansion, reaching a maximum number within 3–4 days post-injury [8,11]. Trophic factors such as IGF1, IL6, Wnt1, Wnt3A and Wnt5A, released from FAPs, provide a transient and stimulatory environment for the activation and differentiation of SCs [8,13]. However, during the remodelling stage of muscle regeneration, these massive FAPs need to be cleared timely from the regenerated areas; unsuccessful FAP clearance impels their differentiation into fibroblasts and adipocytes, resulting in muscle fibrosis and degeneration [11,14]. In addition to acute injuries, dysregulated FAPs are also involved in the aetiological changes of genetic or non-genetic muscular diseases such as Duchenne muscular dystrophy (DMD), neurogenic atrophy and sarcopenia, leading to muscle fibre loss and intramuscular infiltration of fatty or fibrotic tissues [15], [16], [17]. Despite known roles of FAPs in muscle regeneration and degeneration, intrinsic mechanisms controlling their dynamic changes during muscle repair and their fate decision during remodelling remain poorly characterized [10,11,18].

The beneficial effects of retinoic acid (RA) signalling on tissue regeneration has been reported in both cardiac and skeletal muscle [19], [20], [21]. However, the regulatory role of RA-signalling on the cellular states of FAPs has not been reported. As dynamic changes of FAPs are critical for proper muscle regeneration, exploration of the intrinsic mechanisms controlling their cellularity provides opportunities to optimize muscle regeneration process. In this study, we explored the role of retinoic acid receptor (RAR) signalling on cellular states of FAPs and consequent impacts on muscle restoration. Obesity induces multiple adverse changes in skeletal muscle, including metabolic dysfunction, chronic inflammation, and insulin resistance. This disrupts the homoeostasis of the local tissue microenvironment, leading to attenuated myofiber regeneration in addition to increased lipid and collagen accumulation in regenerated skeletal muscle [22], [23], [24], [25], [26]. The contribution of FAPs to obesity-induced regenerative dysfunction has not been examined and possible solutions remain elusive. Therefore, we explored pathological changes of FAPs during impaired regeneration due to obesity and its rescue by RAR activation in FAPs. We found that RAR activation promotes FAP proliferation during the initial stage of muscle regeneration but inhibits their adipogenic and fibrogenic differentiation during the remodelling stage, thus facilitating muscle regeneration. These discoveries have board implications considering that ectopic fatty infiltration and fibrosis are major aetiological factors for a variety of pathological changes and diseases.

## Methods

### Mice

*Tg(Pdgfra-Cre/ERT)467Dbe* (*Pdgfrα-Cre*; RRID: IMSR_JAX:018,280) mice were purchased from the Jackson Laboratory (Bar harbour, Maine). *ROSA26-RARa403* dominant-negative mice were kindly provided by Dr. Cathy Mendelsohn [27]. These two strains were crossbred at Washington State University to generate *Pdgfrα-Cre RARa*403 (RAR*α*DN) mice. Littermates lacking the *cre* allele served as the wide type (WT) group. To verify *Cre*-dependant recombination, *Pdgfrα-Cre* mice were crossbred with Gt(ROSA)26Sor^tm4(ACTB-tdTomato,-EGFP)Luo^ (*ROSA^mT/mG^*, Jackson Laboratory, RRID: IMSR_JAX:007,676) mice to generate *Pdgfrα-Cre ROSA^mT/mG^* mice. All animal studies were conducted in AAALAC-approved facilities and approved by the Institutional Animal Use and Care Committee (IACUC) at Washington State University (Permit No. 06,300).

Lean male mice were fed with a normal fat diet (ND, 10% energy from fat, D12450, Research Diets, New Brunswick, NJ) while obesity was induced by feeding male mice for 12 weeks of a high-fat diet (HFD; 60% energy from fat, D12492; Research Diets) starting at 10 weeks of age. To induce transgenic gene recombination, all mice were intraperitoneally injected with 75 mg/kg tamoxifen (Sigma #T5648) for three days. Two days later, 10 mg/ml of all trans-retinoic acid (RA, Sigma #R2625) dissolved in corn oil or corn oil only was injected subcutaneously above the *Tibialis anterior* (TA) muscle of mice (10 mg/kg body weight) once every other day for 4 days before inducing injury. TA muscle injury was induced by intramuscular injection of 50 µL of 10 µmol/L cardiotoxin (CTX, Sigma #217,503). The experimental sample size was determined by previous studies in our lab [26]. Three mice at a similar age were randomly selected and assigned to each treatment. A total of 72 mice were used for the regeneration studies. Samples were collected at different days post-injury (dpi) to evaluate the regenerating progress.

### Muscle histology

TA muscle was fixed in 4% paraformaldehyde (PFA) and cryopreserved with 30% sucrose before being frozen in isopentane pre-cooled in liquid nitrogen with an embedding OCT compound (Fisher Scientific #23,730,571). Sections were stained for H&E or Masson trichrome staining, and imaging was performed with an EVOS microscope (Advanced Microscopy Group, Bothell, WA, USA) [28]. For immunofluorescence staining, sections were heated in citrate buffer for 20 min and blocked with 1% BSA in TBS containing 0.3% Triton X-100 for 2 h. Slides were incubated overnight at 4 °C with the following primary antibodies: anti-PERILIPIN (Cell Signalling #9349, RRID: AB_10,829,911); anti-COL1α (Santa Cruz #59,772, RRID: AB_1121,787); anti-PDGFRα (R&D Systems #AF1062; RRID: AB_2236,897); anti-cleaved CASPASE 3 (Cell Signalling #9664, RRID: AB_2070,042). Corresponding fluorescent secondary antibodies were applied for 1 h. Nuclei were stained with DAPI in mounting medium (Vector Laboratories #H-1500). Immunofluorescence was imaged using a fluorescence microscope (EVOS FL, Life Technologies). Image J software (NIH) was used to assess size distribution of regenerated myofibers in H&E staining, collagen deposition in Masson trichrome staining, and the ratio of PERILIPIN+ and COL1α+ areas in regenerated TA muscle by fluorescence staining. The percentage of interstitial space was calculated by the percentage of areas absent of muscle fibres in each view. FAP cell numbers were calculated by counting the number of PDGFRα+ cells with nuclei identified by DAPI staining per field. Four representative sections from each muscle sample of different mice from each group were used for measurements.

### Primary cell isolation and purification

Isolation of FAPs was conducted using magnetic activated cell sorting (MACS) following previous reports [10,29]. Briefly, finely minced TA muscles were digested in 800 U/ml Collagenase Ⅱ (Gibco #17,101,015) in Dulbecco modified Eagle medium (DMEM #10,313–021) media for 1 hour at 37 °C. After washing, additional digestion was performed in 100 U/ml Collagenase Ⅱ and 1.1 U/ml Dispase Ⅱ (Gibco #17,105,041) for 30 min. Muscle slurries were then filtered through a 40 μm cell strainer and pelleted at 400 x g. *Re*-suspended cell samples were incubated with anti-CD16/32 antibody (BioLegend # 101,302, RRID:AB_312,801) for 5 min to block Fc receptors. For magnetic isolation, cells were incubated with biotinylated antibodies against anti-CD31 (BioLegend #102,404, RRID: AB_312,899), anti-CD45 (BioLegend #109,804, RRID: AB_313,441), and anti-a7 integrin (Miltenyi Biotec #A130–501–979) followed by incubation with anti-biotin microbeads (Miltenyi Biotec #120–000–900). Cells were loaded on LD columns (Miltenyi Biotec #130–042–901). The flow through fraction was collected and incubated with an anti-Sca1-PE antibody (Biolegend #108,108, RRID: AB_313,345) followed by anti-PE microbeads (Miltenyi Biotec #130–048–801).

### Cell culture

Fresh isolated FAPs (P0) were plated at a density of 1 × 10^4^ cm^2^ in growth media containing DMEM, which was supplemented with 20% heat-inactivated foetal bovine serum (FBS, Gibco #10,439,001), 1% penicillin-streptomycin (Sigma #P0781) plus 2.5 ng/ml of bFGF (Invitrogen #PHG0021). After reaching about 80% confluence, cells were detached (P1) and distributed for subsequent treatments. For adipogenic induction, confluenced FAPs were firstly exposed to adipogenic differentiation medium consisting of DMEM with 20% FBS, 1 μg/mL insulin (Sigma #I3536), 0.5 mM of 3-isobutyl-1-methylxanthine (IBMX, Sigma # I5878), and 1 mM dexamethasone (DEX, Sigma #D4902). Three days later, cells were switched to an adipogenic maintenance medium containing DMEM with 10% FBS and 1 μg/ml insulin for 3 additional days. For fibrotic induction, FAPs were treated with fibrotic induction media consisting of DMEM media with 2% FBS and 2 ng/ml of transforming growth factor b 1 (TGFb1) (PeproTech #100–21) for 5 days. For spontaneous differentiation, FAPs were cultured in growth media for 13 days for adipogenic evaluation or 7 days for fibrotic evaluation. Stocks of RA and BMS493 (Tocris Bioscience #3509) were dissolved in DMSO and diluted by corresponding culture media. To assess FAP proliferation, vehicle only (CON), 1 μM RA, 1 μM BMS493 and combined 1 μM RA with 1 μM BMS493 were added to the growth media of FAPs. Samples were collected one day after treatments and used for immunofluorescence analysis of PCNA.

### Oil red staining

As previously described, differentiated cells were fixed in 4% PFA for 30 min, rinsed with distilled water and 60% isopropanol, and then stained with Oil-Red O (Sigma #O0625) in 60% isopropanol for 10 min [26]. Free dye was removed by washes with distilled water. The percentage of Oil Red O occupied area was quantified and normalized to the control group.

### Immunocytochemical staining

Cells were fixed in 4% PFA for 20 min, permeabilized with 0.1% Triton X-100 for 5 min, blocked with 2% BSA, and incubated with primary antibodies including PERILIPIN (Cell Signalling #9349, RRID: AB_10,829,911) or COL1α (Santa Cruz #59,772, RRID: AB_1121,787) at 4 °C overnight. Alternatively, cells were fixed in cold methanol for 10 min for staining of PCNA (Santa Cruz #25,280, RRID: AB_628,109). Cells were then stained with corresponding secondary antibodies for 1 h. Nuclei were stained with DAPI in mounting medium (Vector Laboratories, Burlingame, CA). Images were taken using a fluorescence microscope (EVOS FL, Life Technologies).

### Quantitative real-time pcr analyses

Total RNA was isolated using TRIzol reagent (Sigma #T9424). The cDNA templates were obtained from 500 ng of purified RNA using iScriptTM cDNA Synthesis kit (Bio-Rad #1708,891). The CFX RT-PCR detection system (Bio-Rad) with a SYBR Green RT-PCR kit (Bio-Rad #1725,274) was used to run qRT-PCR [28]. *β-Actin* (for tissue) or *Gapdh* (for cell) were used as reference genes to normalize mRNA expression levels. Data were analysed using 2-ΔΔCt method [30]. Primer sequences are listed in Table S1. In addition, the cDNA of *RARα403* was amplified to verify its specific transcription in PDGFRα-Cre expressing FAPs using the following primer: Forward 5′-GCGCTCTGACCACTCTCCAGC-3′; Reverse 5′-TGCTTGGCGAACTCCACAGTCTTA-3′ [31].

### Immunoblotting analysis

As previously described, immunoblotting analyses were performed using the Odyssey Infrared Image System (LICOR Biosciences, Lincoln, NE, USA) [32]. The anti-DESMIN (Abcam #15,200, RRID: AB_301,744) and anti-β-ACTIN (Cell signalling, #4967, RRID: AB_330,288) were used as the primary antibodies. The secondary antibodies, IRDye 800CW goat anti-rabbit (#926–32,211, RRID: AB_621,843) and IRDye 680RD goat anti-mouse (#926–68,070, RRID AB_10,956,588), were purchased from LI-COR Biosciences (Lincoln, NE, USA). Quantification of DESMIN expression was normalized according to the expression of β-ACTIN.

### Statistics

Three biological replicates were used for animal and cell culture experiments unless specifically indicated. All data were analysed with GraphPad Prism (version 7) and represented as means ± SEM. All data were found to be normally distributed. For comparison between multiple groups, a one-way ANOVA followed by a Dunnett's multiple comparison were used. For comparison involving both genotype and treatment, a two-way ANOVA followed by Tukey's multiple comparison was used. # shows significant interaction between two factors while & and $ show significant difference between genotypes (WT and RARαDN) and treatments (CON and RA), irrespectively. *<0.05 and **<0.01 show significant difference between two groups.

### Role of funding source

The funding sources were not involved in study design, data collection, data analyses, interpretation, or writing of the report.

## Results

### Loss of retinoic acid signalling in FAPs impairs skeletal muscle regeneration

**Fig. 1:**
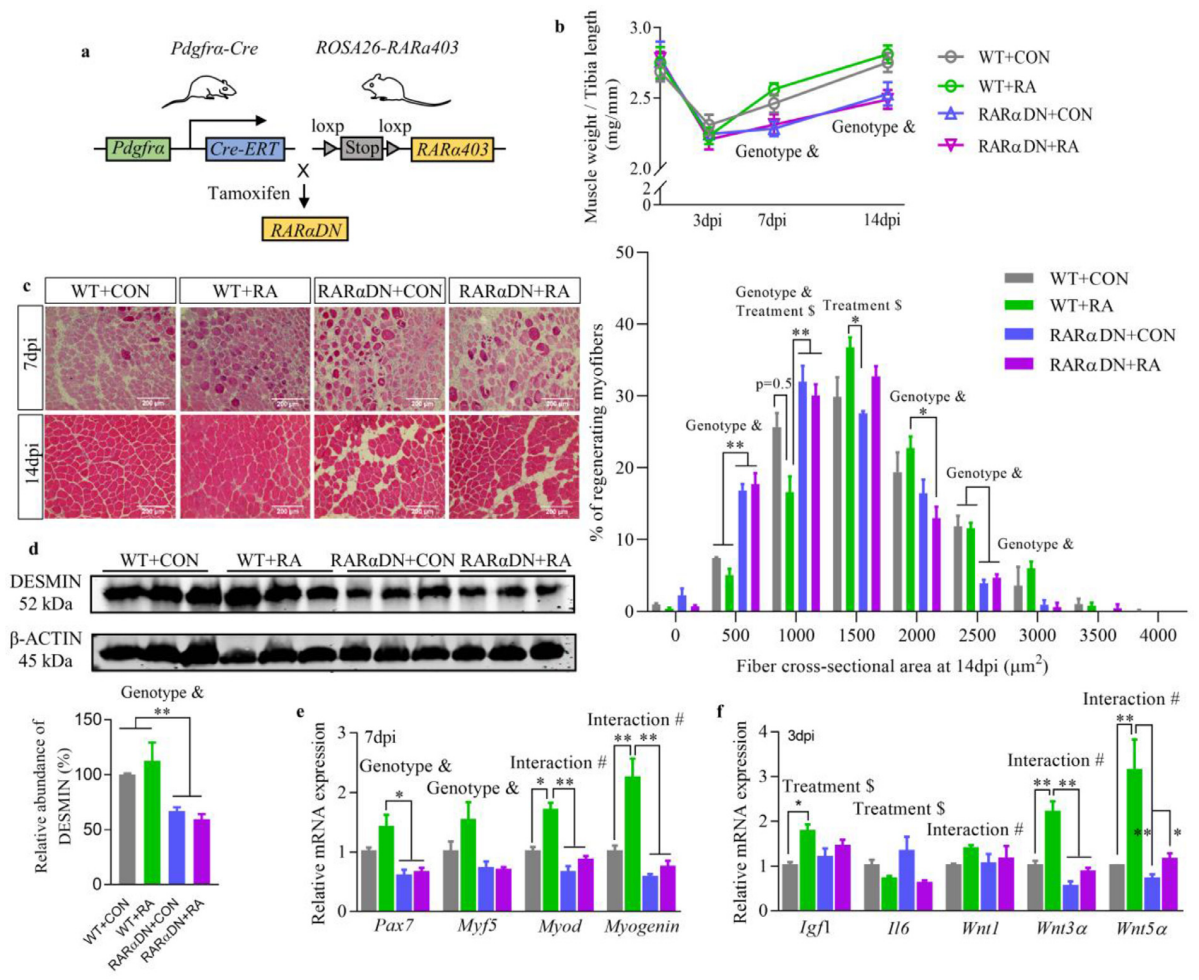
**Loss of retinoic acid (RA) signalling in FAPs impairs skeletal muscle regeneration.** (**a**) A schematic showing dominant negative retinoic acid receptor α (*RARα403*) expression was conditionally induced in PDGFRα-expressing FAPs after administration of tamoxifen and those mice are referred as RARαDN mice. (**b**) Weight of the *Tibialis anterior* (TA) muscle after normalized to tibia length at different days post-injury (dpi). (**c**) H & E staining of regenerated TA muscle and the distribution of cross-sectional areas of regenerated myofibers (fibres with central nuclei) between them. Bars, 200 μm. (**d**) Immunoblotting of DESMIN protein in the regenerated TA muscle at 14 dpi and quantification of its relative abundance after normalized to the expression of β-ACTIN. (**e**) Relative mRNA expression of myogenic genes including *Pax7, Myf5, Myod* and *Myogenin* at 7 dpi. (**f**) Relative mRNA expression of trophic factors for myogenic cells including *Igf1, Il6, Wnt1, Wnt3α* and *Wnt5α* at 3 dpi. Results represent the means ± SEM of three mice per group at each time point. Statistics were analysed using a two-way ANOVA followed by Tukey's multiple comparison. # shows significant interaction (*p*<0.05) between two factors while & and $ show significant difference (*p*<0.05) between genotypes (WT and RARαDN) and treatments (CON and RA), irrespectively. *<0.05 and **<0.01 show significant difference between two groups.

Blockage of RA-signalling in FAPs was achieved through dominant negative expression of a truncated RARα (*RARa403*) specifically in PDGFRα-expressing cells (RARαDN mice, Fig. 1a). This mutant receptor binds with RA but does not induce downstream signalling [27,33]. Cre-dependant recombination was verified using *Pdgfrα-Cre ROSA*^mT/mG^ double fluorescent reporter mice (Fig. S1a). Without Cre expression, cell membrane-localized tdTomato (mT) fluorescence was widely expressed in all cells of the skeletal muscle (Fig. S1b). After tamoxifen-induced recombination, EGFP fluorescence (mG) replaced the expression of tdTomato in cells located in the interstitial space between myofibers, which is consistent with the location of FAPs. To confirm the specific transcription of *RARα403* in FAPs, freshly isolated FAPs and any remaining cells (Non-FAPs) from the TA muscle of WT and RAR*α*DN mice were used for RT-PCR analysis (Fig. S1c). While *RARα403* was detected in FAPs isolated from RAR*α*DN mice, it was hardly detectable in other types of cells including remaining cells (Non-FAPs) from RAR*α*DN and WT mice. Furthermore, the expression of RA-responsive genes including *Crbp1, Cyp26a1, Rarα, Rarβ*, and *Rarγ* were decreased only in FAPs from RAR*α*DN mice (Fig. S1d). Overall, these data verified that RA-signalling was specifically suppressed in FAPs of RARαDN mice.

Loss of RA-signalling in FAPs resulted in lower muscle weight factored for tibia length in RARαDN groups compared to WT groups at both 7 and 14 days-post injury (dpi; Fig. 1b). At 14 dpi, H&E staining showed well-restored muscle structure in both WT+CON and WT+RA groups (Fig. 1c), while substantial interstitial space between myofibers (areas absent of myofibers) was observed in the RARαDN+CON group compared to the WT+CON group (Fig. S2a). Significant interactions between genotypes and treatments (*p*<0.05, two-way ANOVA) on the percentage of interstitial space suggest important roles of RA-signalling in FAPs for the formation of non-myogenic areas in regenerated muscle. In addition, regenerated myofibers in RARαDN groups were smaller, as shown by higher percentages of fibres between 0–250 and 250–750 μm^2^ at 7 dpi, and between 250–750 and 750–1250 μm^2^ at 14 dpi (Fig. S2b and Fig. 1c). RA treatment increased myofiber sizes regardless of genotypes. However, no significant interactions (*p*>0.05, two-way ANOVA) were found between genotypes and treatments, suggesting FAPs-independent roles of RA treatment in myofiber regeneration. Impaired myofiber regeneration in the RARαDN groups was also confirmed by a decreased abundance of DESMIN in regenerated skeletal muscle at 14 dpi compared to WT groups (Fig. 1d). In addition, we also evaluated the number of SCs in the skeletal muscle following RA injection but before inducing injury (Fig. S2c). Two days after the last RA injection, SC density was higher in the RA-treated groups compared to the groups without RA treatment. The direct stimulation on SC proliferation due to RA could partially explain the increased size of the regenerating myofibers after RA supplementation in both groups (Fig. 1c, Fig. S2b, Fig. S2c).

We further evaluated the expression of myogenic markers at earlier stages after injury (Fig. S2d). RA treatment increased *Pax7* and *Myod* expression at 3 dpi and *Myod* and *Myogenin* expression at 7 dpi of WT mice while this increasement was interrupted by the RA-signalling blockage in FAPs (Fig. 1e and Fig. S2d). On the other hand, loss of RA-signalling in FAPs down-regulated *Myod* expression at 3 dpi and *Pax7* and *Myf5* expression at 7 dpi compared to WT groups. In addition, RA treatment also up-regulated gene expression of trophic factors including *Igf1, Wnt3α* and *Wnt5α* of WT mice at 3 dpi, while RA-induced expression of *Wnt3α* and *Wnt5α* was blocked in RARαDN+RA groups (Fig. 1f). These data showed that RA-signalling in FAPs is needed for mediating myogenesis at the early stage of regeneration. In summary, loss of RA-signalling in FAPs impairs myogenesis during the initial stage of muscle regeneration, thus impairing muscle fibre regeneration.

## Retinoic acid signalling suppresses adipogenic differentiation of FAPs

**Fig. 2:**
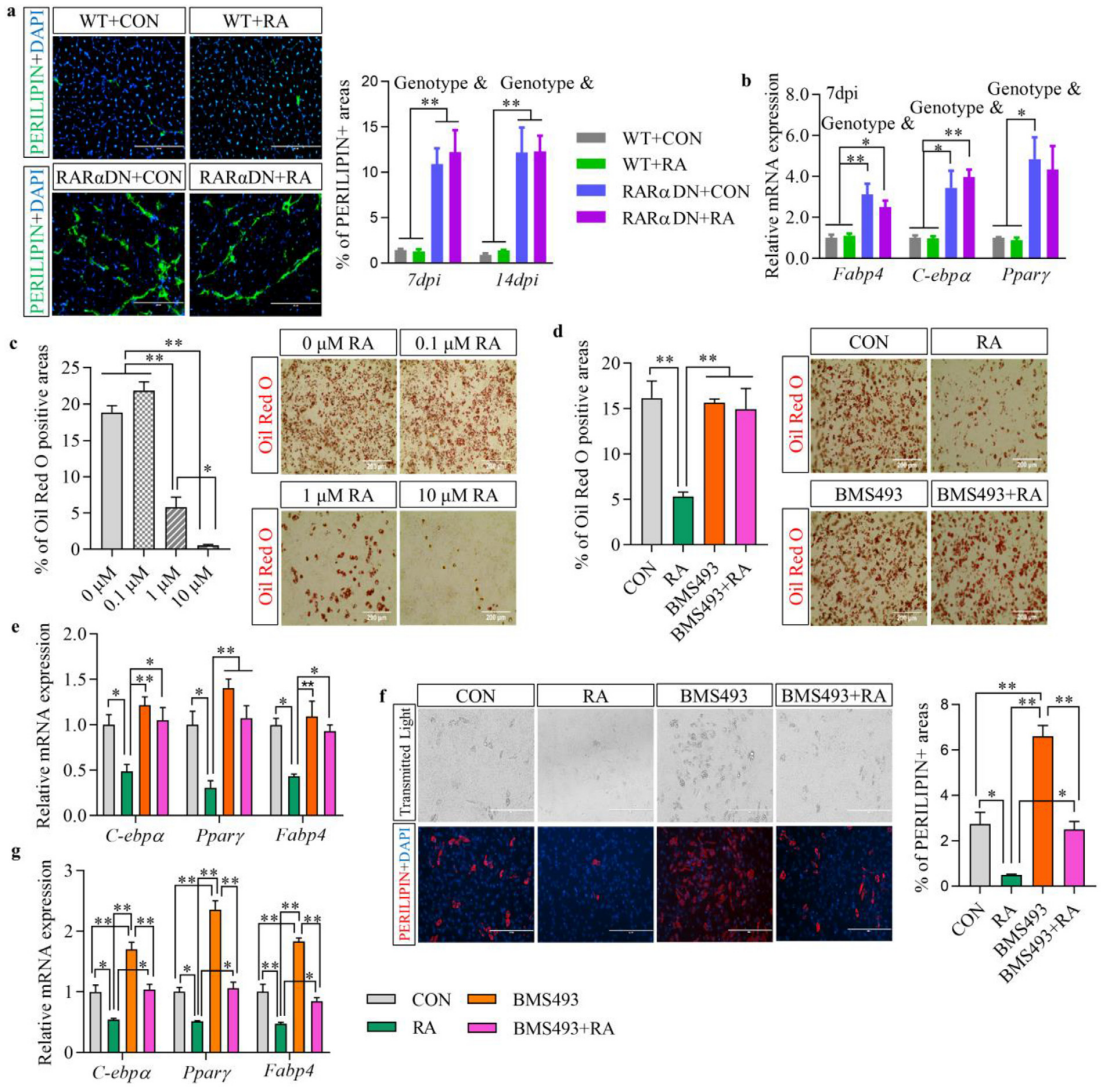
**Retinoic acid (RA) signalling suppresses the adipogenic differentiation of FAPs.** (**a**) Immunofluorescence analysis of PERILIPIN expression at 14 days post-injury (dpi) and the percentage of PERILIPIN+ areas in regenerated skeletal muscle at 7 and 14 dpi. (**b**) Relative mRNA expression of adipogenic markers including *Fabp4, C-ebpα* and *Pparγ* in regenerated skeletal muscle at 7 dpi. (**c**) Oil Red O staining of isolated FAPs and percentage of Oil Red O positive areas after adipogenic induction with different doses of RA (0, 0.1, 1 and 10 μM) treatment. (**d**) Oil Red O staining of isolated FAPs and percentage of Oil Red O positive areas after adipogenic induction with treatments of CON (vehicle only), 1 μM RA, 1 μM BMS493, and combined 1 μM RA + 1 μM BMS493. (**e**) Relative mRNA expression of adipogenic markers including *Fabp4, C-ebpα* and *Pparγ* in isolated FAPs after adipogenic induction with treatments of CON, RA, BMS493 or combined RA + BMS493. (**f**) Immunofluorescence analysis of PERILIPIN expression and percentage of PERLIPIN+ areas in isolated FAPs after cultured in growth media for 13 days. (**g**) Relative mRNA expression of adipogenic markers including *C-ebpα, Pparγ* and *Fabp4* in isolated FAPs after cultured in growth media for 13 days. Bars, 200 μm. Results represent the means ± SEM of three mice per group at each time point. Statistics were analysed using a two-way (**a, b**) or one-way ANOVA (**c**-**g**). # shows significant interaction (*p*<0.05) between two factors while & and $ show significant difference (*p*<0.05) between genotypes (WT and RARαDN) and treatments (CON and RA), irrespectively. *<0.05 and **<0.01 show significant difference between two groups.

To further characterize fat infiltration in RARαDN and RARαDN+RA groups shown by H&E staining (Fig. 1c), the presence of adipocytes was further confirmed by PERILIPIN expression (Fig. 2a and Fig. S2e). Loss of RA-signalling in FAPs caused ectopic adipocyte formation at both 7 and 14 dpi, which was not prevented due to RA treatment of RARαDN mice. Consistently, loss of RA-signalling in FAPs induced higher expression of adipogenic markers including *Fabp4, C-ebpα*, and *Pparγ* at 7 dpi, which was not observed in the presence of RAR-signalling (Fig. 2b). Because CTX-induced injury does not induce adipocyte formation in WT lean mice, very few PERILIPIN-expressing adipocytes were detected in WT groups in this study and supplementation of RA did not further decrease that (Fig. 2a and Fig. S2e) [34]. As a result, no interactions on adipogenesis were found between genotypes and treatments.

To validate the effects of RA-signalling on the adipogenic differentiation of FAPs, we isolated FAPs from the lower limb muscle of WT mice. After adipogenic induction by culturing FAPs in adipogenic medium which drives adipogenic differentiation, large areas of lipid droplets were detected in FAPs without RA treatment, which was dose-dependently inhibited by RA (Fig. 2c). Both 1 and 10 μM RA dramatically suppressed fat formation, and 1 μM RA was chosen for further studies. BMS493, an antagonist of the pan-RARs, was used to inhibit RA-signalling in isolated WT FAPs. Addition of RA to the adipogenic media reduced fat formation compared to the CON group, however, inhibition of RA-signalling by BMS493 did not affect adipocyte formation (Fig. 2d). As large areas of adipocytes were found in the CON group in which FAPs might reach their maximal adipogenic potential driven by the adipogenic medium, BMS493 did not further increase adipogenesis. Combined treatment of RA and BMS493, which neutralizes RAR, blocked the inhibitory role of RA on adipocyte formation. Consistently, expression of adipogenic genes including *C-ebpα, Pparγ* and *Fabp4* were reduced in the RA-treated group compared to the CON group, while their expression was reversed in the BMS493+RA group (Fig. 2e). In addition, we investigated spontaneous adipogenic differentiation of FAPs in growth media for 13 days (Fig. 2f). Without adipogenic stimulation, adipogenic differentiation of FAPs is spontaneous. Consistently, RA treatment reduced areas of PERILIPIN-expressing adipocytes compared to the CON group. Moreover, blockage of RAR-signalling in FAPs promoted formation of PERILIPIN-expressing adipocytes, which was blocked in BMS493+RA group. Meanwhile, RA treatment inhibited, while BMS493 treatment promoted, expression of adipogenic genes including *C-ebpα, Pparγ* and *Fabp4* compared to the CON group during spontaneous differentiation (Fig. 2g). Therefore, adipogenesis of FAPs is inhibited by RA-signalling in FAPs.

## Retinoic acid supplementation inhibits fibrogenic differentiation of FAPs

**Fig. 3:**
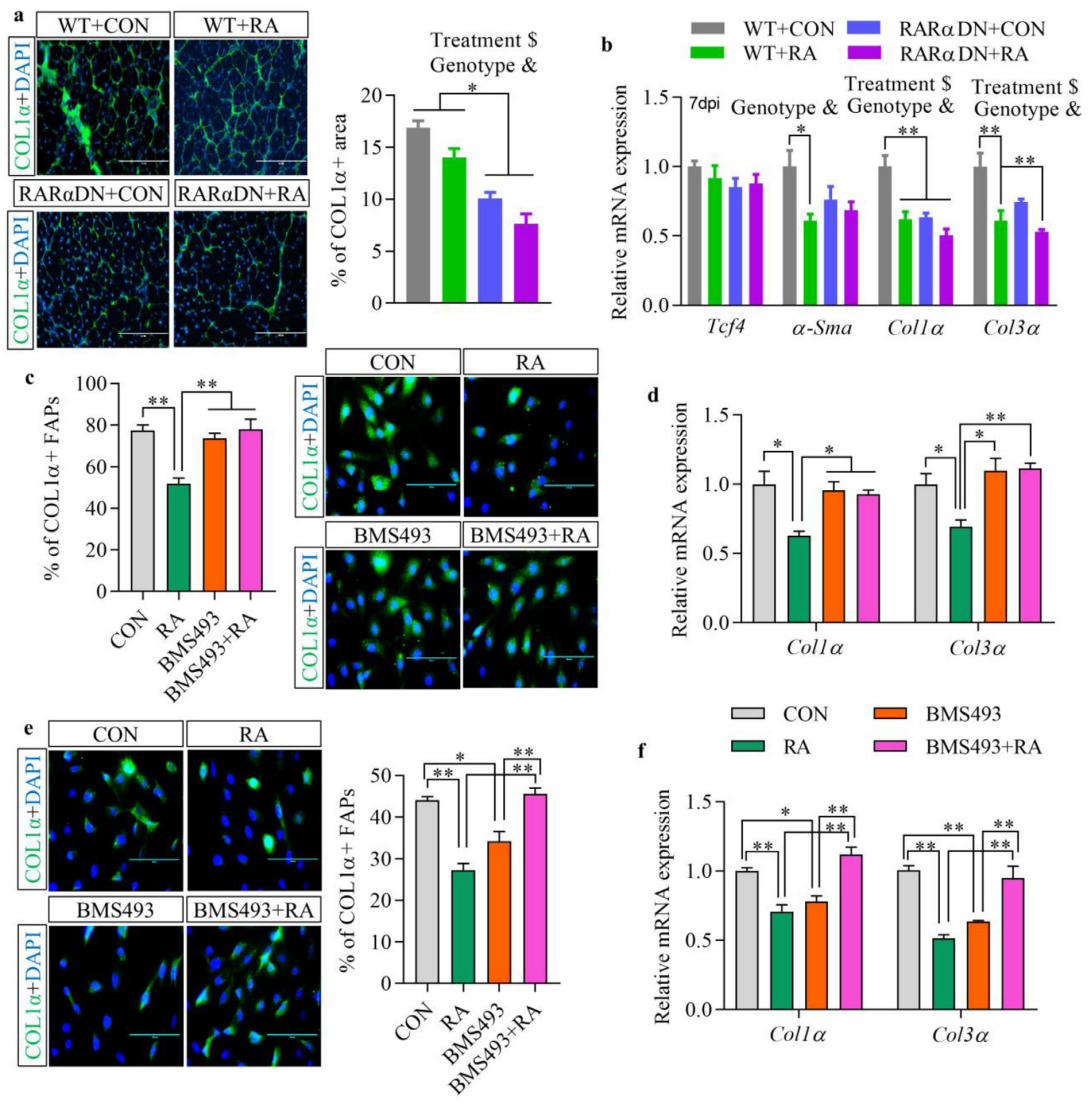
**Retinoic acid signalling inhibits fibrotic differentiation of FAPs.** (**a**) Immunofluorescence images of COL1α and percentage of COL1α+ areas in regenerated muscle at 14 days post-injury (dpi). Bars, 200 μm. (**b**) Relative mRNA expression of fibrogenic markers including *Tcf4, α-Sma, Col1α* and *Col3α* in regenerated muscle at 7 dpi. (**c**) Immunofluorescence images of COL1α and percentage of COL1α+ FAPs after fibrotic induction of FAPs *in vitro*. Bars, 100 μm. (**d**) Relative mRNA expression of *Col1α* and *Col3α* in isolated FAPs after fibrotic induction of FAPs *in vitro*. (**e**) Immunofluorescence images of COL1α and percentage of COL1α+ FAPs after *in vitro* culturing FAPs in growth media for 7 days. Bars, 100 μm. (**f**) Relative mRNA expression of *Col1α* and *Col3α* after *in vitro* culturing FAPs in growth media for 7 days. Results represent the means ± SEM of three mice per group at each time point. Statistics were analysed using a two-way (**a, b**) or one-way ANOVA (**c**-**f**). # shows significant interaction (*p*<0.05) between two factors while & and $ show significant difference (*p*<0.05) between genotypes (WT and RARαDN) and treatments (CON and RA), irrespectively. *<0.05 and **<0.01 show significant difference between two groups.

The effects of RA-signalling on fibrotic tissue formation in regenerated skeletal muscle were studied by immunofluorescence staining of COL1α at 14 dpi (Fig. 3a). Supplementation of RA reduced COL1α+ areas in regenerated muscle regardless of genotypes. Decreased fibrogenic gene expression of *Col1α* and *Col3α* was also found at 7 dpi in RA-treated groups (Fig. 3b). No interactions between the treatment and genotype were found on COL1α+ areas and fibrotic gene expression (Fig. 3a and Fig. 3b). These results suggest that the observed fibrosis reduction caused by the RA treatment was independent of RA-signalling in FAPs. Interestingly, blockage of RA-signalling in FAPs decreased fibrotic tissue accumulation in RARαDN groups at 14 dpi, which was consistent with down-regulated gene expression of *α-Sma, Col1α* and *Col3α* at 7 dpi compared to WT groups (Fig. 3a and Fig. 3b).

Effects of RA-signalling on fibrotic differentiation of FAPs were also investigated *in vitro*. After culturing FAPs in a fibrotic-induction medium with TGFb1, the percentage of COL1α positive FAPs was lower in RA treatment group with decreased gene expression of *Col1α* and *Col3α*, while no difference was found in the BMS493 treatment group compared with the CON group (Fig. 3c and d). These data showed that the loss of RA-signalling does not change fibrotic differentiation of FAPs under a fibrogenic environment. When FAPs were cultured in growth media without fibrogenic induction and differentiated spontaneously, treatment with RA reduced the percentage of COL1α-expressing FAPs (Fig. 3e). Surprisingly, inhibition of RA-signalling also reduced COL1α expression, which should be due to a shift from fibrogenesis to adipogenesis of FAPs in the absence of RA-signalling (Fig. 2f). Treatment of RA and BMS493 to FAPs at the same time, which neutralized RA-signalling, did not affect the spontaneous differentiation of FAPs compared to CON group. Gene expression of *Col1α* and *Col3α* also showed consistent changes (Fig. 3f). Overall, these data suggest that supplementation of RA inhibits the fibrogenic differentiation of FAPs both *in vivo* and *in vitro* while blockage of RA-signalling in FAPs shifts their differentiation to adipogenic, rather than fibrogenic.

**Fig. 4:**
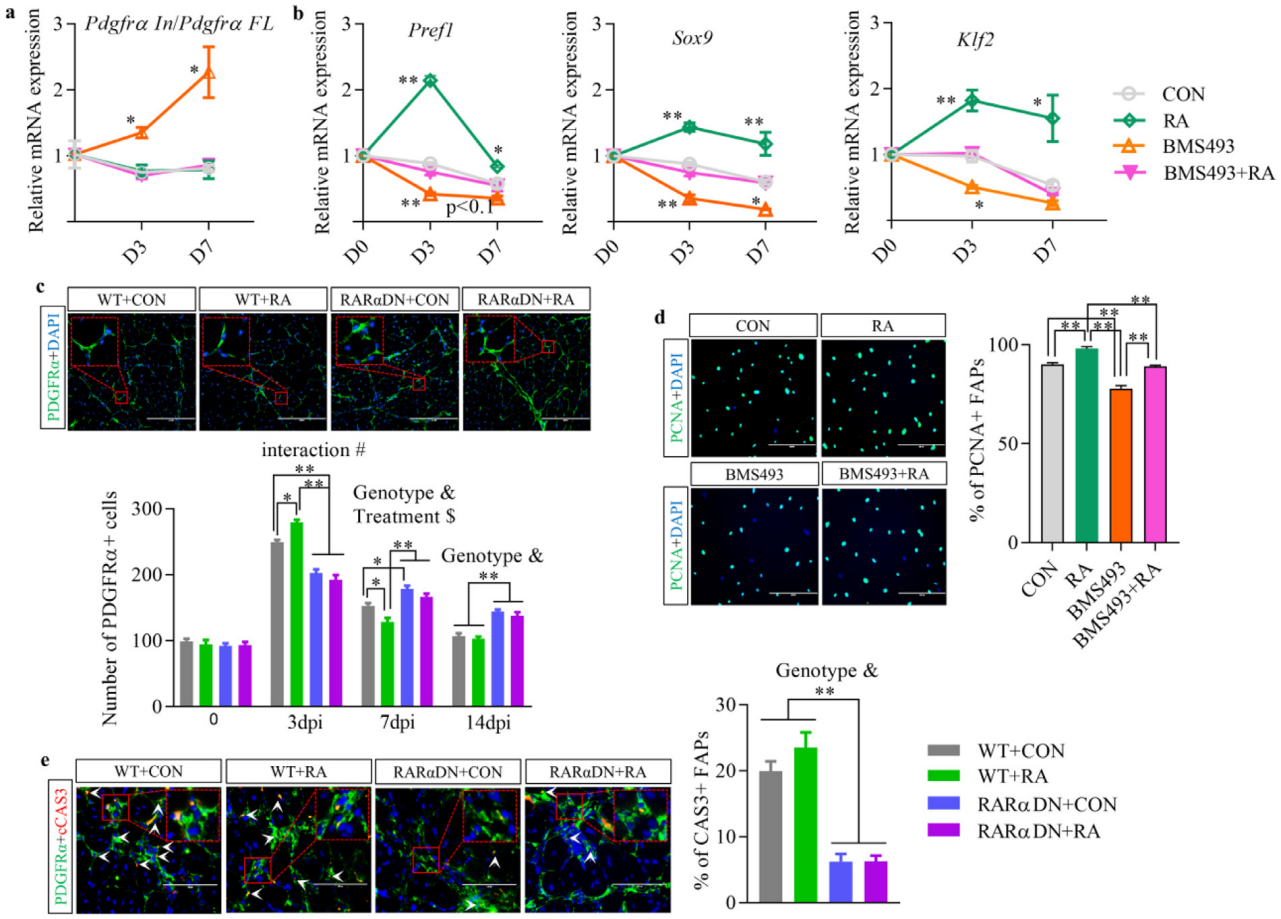
**Retinoic acid regulates cellularity of FAPs.** (**a**) Relative mRNA expression of the ratio of intronic variant (In) to full-length (FL) *Pdgfrα* transcripts (*Pdgfrα In*/*Pdgfrα FL*) during *in vitro* culture of confluent FAPs in growth media for 7 days. (**b**) Relative mRNA expression of preadipocyte genes including *Pref1, Sox9* and *Klf2* during *in vitro* culture of confluent FAPs in growth media for 7 days. (**c**) Immunofluorescence analysis of PDGFRα in regenerated TA muscle at 14 days post-injury (dpi) and quantification of the number of PDGFRα+/DAPI+ FAPs per field at different times post-injury. Bars, 200 μm. (**d**) Immunofluorescence analysis of PCNA and percentage of PCNA+/DAPI+ FAPs at 24 h after different treatments. Bars, 200 μm. (**e**) Immunofluorescence analysis of cleaved CASPASE3 (cCAS3) and quantifications of the percentage of cCAS3+ FAPs in regenerated muscle at 7 dpi. Bars, 100 µm. Results represent the means ± SEM of three mice per group at each time point. Statistics were analysed using a one-way (**a, b**, and **d**) or two-way (**c, e**) ANOVA. # shows significant interaction (*p*<0.05) between two factors while & and $ show significant difference (*p*<0.05) between genotypes (WT and RARαDN) and treatments (CON and RA), respectively. *<0.05 and **<0.01 show significant difference between two groups. For (**a**) and (**b**), significant difference compared to CON group was labelled.

To elucidate regulatory mechanisms of RA-signalling on FAP cellularity, we evaluated associated gene expression. Alternative processing of the *Pdgfrα* transcripts were reported to regulate adipogenic or fibrogenic fate decision of FAPs, with an increased ratio of intronic variant of *Pdgfrα* (*Pdgfrα In*) to full length of *Pdgfrα* (*Pdgfrα FL*) associated with decreased fibrogenic potential [9]. A gradually increased ratio of *Pdgfrα In*/*Pdgfrα FL* was found when BMS493 was added to the growth media of FAPs *in vitro*, however, RA supplementation did not change this ratio (Fig. 4a). Therefore, alternative processing of the *Pdgfrα* transcripts might not be involved in the RA-induced inhibition on FAP differentiation. Thus, we further investigated the expression of preadipocyte genes including *Pref1, Sox9* and *Klf2*, which are direct targets of RAR (Fig. 4b). Their expression was increased in the RA group while decreased in the BMS493 group. Increased preadipocyte gene expression in adipogenic progenitors is associated with inhibited adipogenic differentiation [35]. In summary, RA-signalling inhibits the adipogenic differentiation of FAPs.

## Retinoic acid regulates cellularity of FAPs

To elucidate the mechanism of RA-signalling in FAPs-induced changes during muscle regeneration, the chronological changes of FAPs during this process were investigated. Supplementation of RA to WT mice increased the proliferation of FAPs at 3 dpi while blockage of RA-signalling reduced their proliferation in the RARαDN+CON group compared to the WT+CON group (Fig. S2f and Fig. 4c). Significant interaction (*p*<0.01, two-way ANOVA) between genotypes and treatments was found on the proliferation of FAPs at 3 dpi, suggesting important roles of RA-signalling in FAPs for muscle regeneration. In addition, the percentage of PCNA+ FAPs was also increased in the RA-treated group and reduced in the BMS493-treated group while treatment of RA and BMS493 simultaneously diminished RA-induced proliferation (Fig. 4d). Though increased proliferation of FAPs at an early stage is beneficial for myogenesis, failed clearance of FAPs at a later stage leads to excessive adipogenesis or fibrosis. A higher number of FAPs remained in regenerated skeletal muscle of RARαDN groups at both 7 and 14 dpi compared to WT groups (Fig. S2f and Fig. 4c). Also, loss of RA-signalling in RARαDN groups reduced the proportion of cleaved CASPASE3+ (cCAS3+) FAPs compared to WT groups at 7 dpi (*p*<0.01), regardless of RA treatment (Fig. 4e). No interaction between genotypes and treatments was found on the number of FAPs at 7 and 14dpi, and the number of cCAS3+ FAPs at 7 dpi, suggesting the involvement of other types of cells in the regulation of FAP apoptosis. In summary, loss of RA-signalling impairs the proliferation of FAPs at an early stage while reducing their apoptosis at the remodelling stage, resulting in impaired muscle regeneration.

## Supplementation of ra rescues skeletal muscle regeneration impaired due to obesity

Chronic inflammation is associated with declined RA-signalling in tissues while modest decreases in endogenous RA levels are reported to enhance adipogenic differentiation [36], [37], [38], [39]. Since obesity is also characterized with an increased inflammatory response, we further explored the expression of RA signals in the skeletal muscle of obese mice and the effects of RA-signalling in FAPs for muscle regeneration. We firstly compared the expression of RA-signalling responsive genes in lean and high-fat-diet induced obese mice (Fig. S3a). While the expression of *Rarβ, Rxrγ* and *Aldh1α1* was up-regulated in obese mice, expression of *Rarγ, Aldh1α2, and Cyp26b1* was down-regulated. We also analysed the expression of *Crabp1, Crabp2* and *Cyp26a1*, which was hardly detectable. Consistently, as a feedback mechanism, the attenuation of RA signals increases the expression of RA-synthesizing enzymes (retinaldehyde dehydrogenases, ALDH1As) while decreases expression of RA-catabolizing enzymes (CYP26A1 and CYP26B1) [40], [41], [42]. Therefore, our finding suggest overall declined RA-signalling in the skeletal muscle of obese mice.

**Fig. 5:**
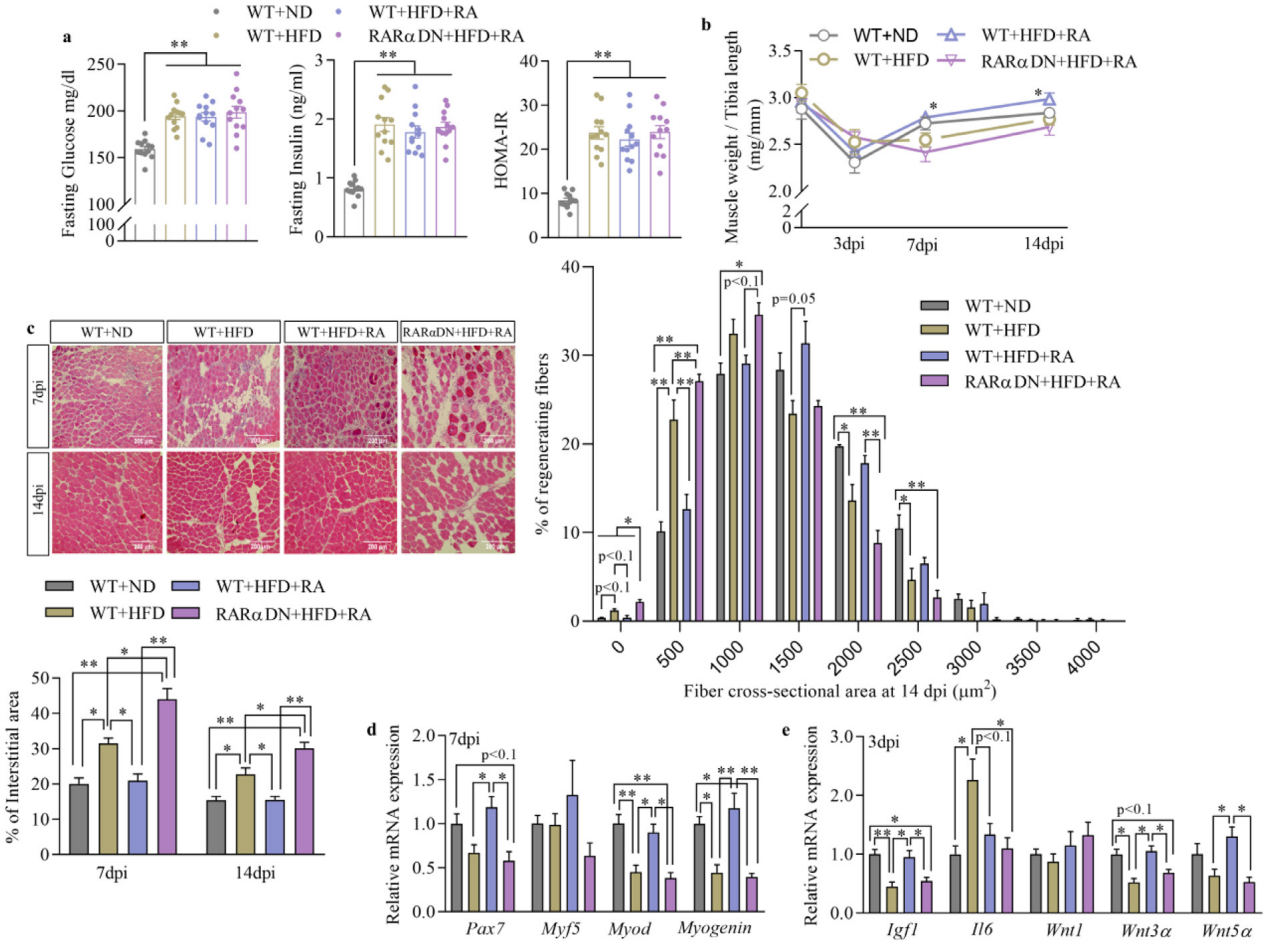
**Supplementation of RA rescues skeletal muscle regeneration impaired due to obesity.** (**a**) Blood glucose, insulin and calculated HOMA-IR after 5 h of fasting. *n* = 12. (**b**) Weight of regenerated *Tibialis anterior* (TA) muscle after normalized to tibia length at different days post-injury (dpi). (**c**) H & E staining of regenerated skeletal muscle, and distribution of cross-sectional areas of regenerated myofibers (fibres with central nuclei) and percentage of interstitial areas between them. Bars, 200 μm. (**d**) Relative mRNA expression of myogenic genes including *Pax7, Myf5, Myod* and *Myogenin* at 7 dpi. (**e**) Relative mRNA expression of trophic factors including *Igf1, Il6, Wnt1, Wnt3α* and *Wnt5α* at 3 dpi. Results represent the means ± SEM of three mice per group at each time point. Statistics were analysed using a one-way ANOVA. *<0.05 and **<0.01 show significant difference between two groups. For (**b**), significant difference compared to WT+ND group was labelled.

Regeneration of skeletal muscle in obese mice is characterized with excessive fat and fibrotic infiltration [24,25]. To investigate the effects of RA-signalling in FAPs for muscle regeneration in obese mice, we treated HFD-induced obese WT and RARαDN mice with/without RA and compared their regeneration process. The HFD treatment induced body weight gain along with elevated fasting glucose, insulin, and HOMA-IR (Fig. 5a and Fig. S3b). Following CTX-induced injury, no difference in TA muscle weight factored to Tibia length was found between lean and obese mice (Fig. 5b). Regenerated TA muscle weight was higher in WT+HFD+RA group than in the RARαDN+HFD+RA group at both 7 and 14 dpi. Obesity impairs muscle regeneration as shown in H&E staining with more interstitial areas compared to the lean mice at both 7 and 14 dpi (Fig. 5c). Supplementation of RA reduced interstitial spaces in the regenerated TA muscle of WT obese mice, but not in obese mice without RA-signalling in FAPs. The size distribution of regenerated myofibers in WT obese group decreased compared to CON mice (Fig. S3c and Fig. 5c). Supplementation of RA reversed those changes; however, these beneficial effects were blocked in obese mice with RA-signalling blockage in FAPs. In addition, the HFD-treatment reduced expression of myogenic genes including *Pax7, Myf5* and *Myod* at 3 dpi*,* and *Myod* and *Myogenin* at 7 dpi, which was largely rescued by RA supplementation to WT obese mice but not to RARαDN obese mice (Fig. 5d and Fig. S3d). Decreased expression of *Igf1* and *Wnt3a* was also found in obese mice compared to lean mice (Fig. 5e). While supplementation of RA rescued decreased *Igf1, Wnt3α* and *Wnt5α* expression in obese mice at 3 dpi, this beneficial effect was blocked in the absence of RA-signalling in FAPs of obese mice. Therefore, supplementation of RA rescues muscle regeneration impaired due to obesity through stimulating RA-signalling in FAPs.

## Supplementation of ra inhibits both adipogenesis and fibrogenesis in regenerated skeletal muscle of obese mice

**Fig. 6:**
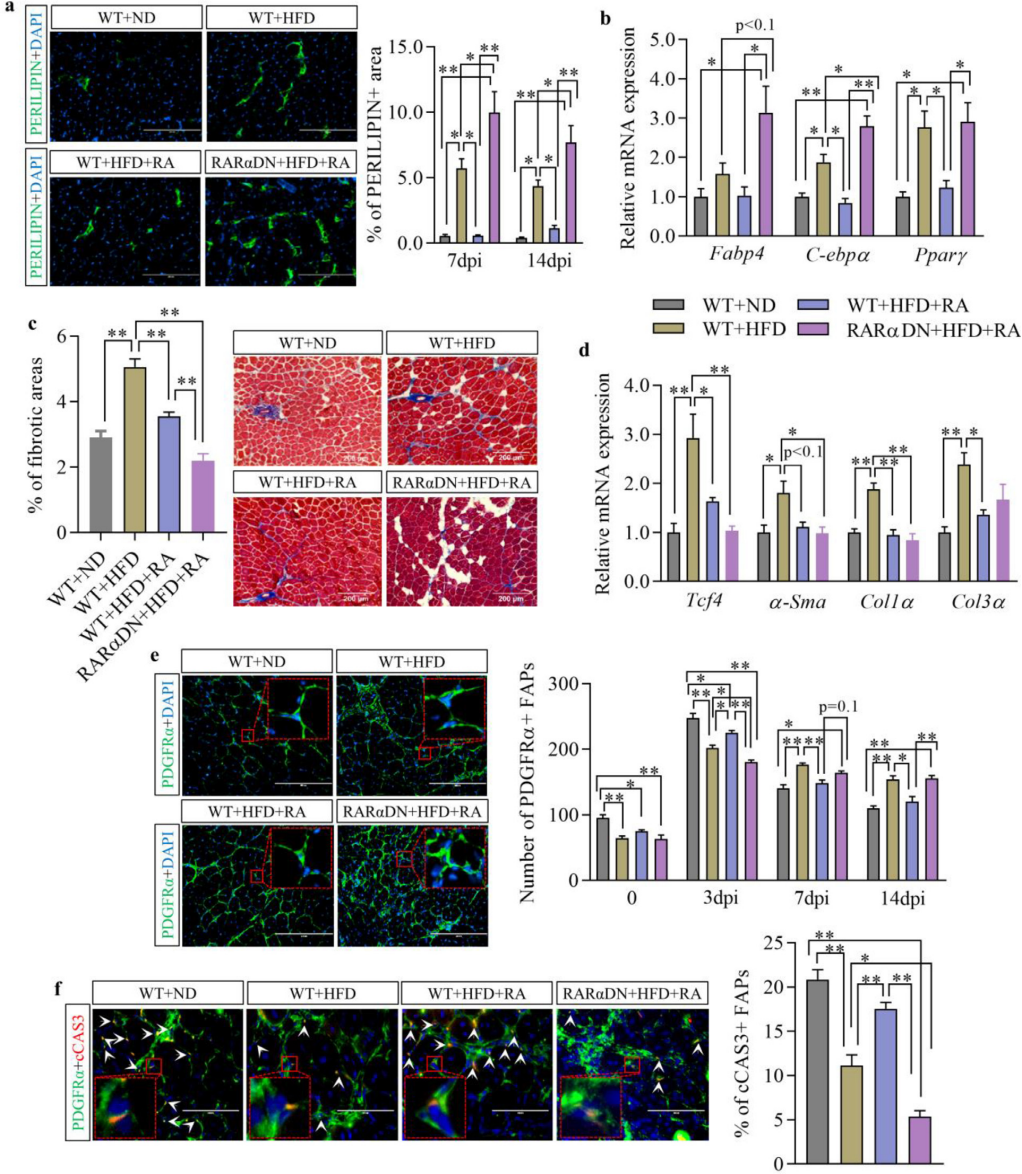
**Supplementation of RA inhibits both adipogenesis and fibrosis in the regenerated skeletal muscle of obese mice.** (**a**) Immunofluorescence analysis of PERILIPIN and the percentage of PERILIPIN+ areas in regenerated muscle at 14 days post-injury (dpi). Bars, 200 μm. (**b**) Relative mRNA expression of adipogenic markers including *Fabp4, C-ebpα* and *Pparγ* in regenerated skeletal muscle at 7 dpi. (**c**) Masson trichrome staining of regenerated muscle at 14 dpi and percentage of fibrotic areas per field. Bars, 200 μm. (**d**) Relative mRNA expression of fibrogenic markers including *Tcf4, α-Sma, Col1α* and *Col3α* in regenerated TA muscle at 7 dpi. (**e**) Immunofluorescence analysis of PDGFRα in regenerated TA muscle at 14 days post-injury (dpi) and quantification of the number of PDGFRα+/DAPI+ FAPs per field at different times post-injury. Bars, 200 μm. (**f**) Immunofluorescence analysis of cleaved CASPASE3 (cCAS3) positive FAPs and quantifications of the percentage of cCAS3+ FAPs. Bars, 100 μm. Results represent the means ± SEM of three mice per group at each time point. Statistics were analysed using a one-way ANOVA. *<0.05 and **<0.01 show significant difference between two groups.

Obesity increased areas of PERILIPIN-expressing adipocytes in regenerated skeletal muscle compared to lean mice at both 7 and 14 dpi, which was inhibited by RA treatment (Fig. 6a and Fig. S3e). Blockage of RA-signalling in FAPs abolished the inhibitory effects of RA on adipogenesis in obese mice. Consistently, expression of adipogenic genes including *C-ebpα* and *Pparγ* at 7 dpi was increased in obese mice compared to lean mice (Fig. 6b). Supplementation of RA inhibited adipogenic gene expression in WT obese mice but not in obese mice without RA-signalling in FAPs. In addition, Masson trichrome staining showed increased fibrotic tissue accumulation in regenerated skeletal muscle of obese mice at 14 dpi, which was inhibited by RA treatment (Fig. 6c). Blockage of RA-signalling in FAPs of obese mice further decreased fibrotic tissue accumulation compared to the other groups, explained by their enhanced fatty infiltration (Fig. 6a). Obesity also increased the expression of fibrotic genes including *Tcf4, α-Sma, Col1α* and *Col3α* compared to lean mice at 7 dpi, while expression of *Tcf4, Col1α* and *Col3α* was inhibited after RA supplementation (Fig. 6d).

Dynamic changes of FAPs were tracked by immunofluorescent staining of PDGFRα-expressing cells before and at different times post-injury (Fig. 6e and Fig. S3f). The number of PDGFRα-positive FAPs was significantly lower (*p*<0.01) in the skeletal muscle of obese mice before injury and they failed to proliferate sufficiently at 3 dpi compared to lean mice. Failed clearance of FAPs in the regenerated muscle of obese mice was also found at 7 and 14 dpi with a higher number of surviving FAPs (Fig. 6e and S3f). Supplementation of RA to obese mice not only rescued impaired proliferation of FAPs at 3 dpi, but also promoted their timely clearance in the regenerated muscle at 7 and 14 dpi. Blockage of RA-signalling in FAPs of obese mice abolished beneficial effects of RA on proliferation at 3 dpi and the clearance of FAPs at 14 dpi. Consistently, the proportion of FAPs undergoing apoptosis (cleaved CASPASE3+) was lower in regenerating muscle of obese mice at 7 dpi (Fig. 6f), but elevated due to RA supplementation while loss of RA-signalling in FAPs abolished such effects. In summary, obesity impairs skeletal muscle regeneration by attenuating FAP proliferation at an early stage and timely clearance at a later stage. RA supplementation rescues skeletal muscle regeneration impaired due to obesity through promoting FAP proliferation during the initial stage and apoptosis of FAPs at a later stage of muscle regeneration.

## Discussion

Since FAPs were identified, the supportive functions of FAPs on myogenesis during muscle regeneration have attracted considerable attentions [8,12]. Trophic factors including IGF1, IL-6, Wnt1, Wnt3A and Wnt5A released by FAPs promote proliferation and differentiation of SCs, while the disruption of FAPs causes attenuated myogenesis and muscle loss under pathological conditions [8,43,44]. Our study showed that RA-signalling in FAPs is indispensable for the proliferation of FAPs at an early stage of regeneration along with the expression of *Wnt1, Wnt3α* and *Wnt5α*. One recent study also showed that FAPs are the primary source of WNT ligands in skeletal muscle, which is important for their pro-myogenic functions [13]. Meanwhile, impaired proliferation and release of trophic factors were also found in obese FAPs, and supplementation of RA rescues this disrupted function of FAPs. Noticeably, RA treatment significantly increased the sizes of regenerated myofibers in obese mice where regeneration process was inhibited. These data show that RA-signalling in FAPs promotes myogenesis and muscle regeneration. In this study, we focus on FAPs, yet we should also consider the direct role of RA on other cell types such as satellite cells, which can also be affected by RA-supplementation [45,46]. Consistently, we found that SC-proliferation was stimulated by RA injection before muscle injury, which should also contribute to the improved muscle regeneration.

FAPs mostly maintain quiescence *in vivo* in healthy skeletal muscle, but they spontaneously differentiate into adipocytes or fibroblasts when cultivated *in vitro* [8,12]. The differentiation of FAPs is thus speculated to be tightly regulated by microenvironment in the skeletal muscle. Altered microenvironment due to pathological conditions induces ectopic adipogenic or fibrogenic differentiation of FAPs [11,15,47]. In our study, we found adipogenesis of FAPs was inhibited by RA-signalling both *in vivo* and *in vitro*. A recent study found that RA directly induced the expression of preadipocyte genes including *Pref1, Sox9* and *Klf2* in isolated preadipocytes from adipose tissue, which suppressed adipogenesis [35]. Consistently, we also found that expression of preadipocyte genes was increased after treating FAPs with RA, which maintains FAPs in a preadipocyte state and restricts their differentiation. In short, RAR-signalling directly inhibits adipogenic differentiation and promotes FAPs to maintain an undifferentiated state.

Transitions of FAPs from proliferation, differentiation, and apoptosis are well controlled by the dynamic niche in the skeletal muscle after injury. Inflammatory cells such as eosinophils and macrophage are recruited immediately after injury, to form a transitional niche for the activation and proliferation of FAPs via the release of cytokines such as IL-4 [10,48]. In absence of these proliferative cytokines, FAPs fail to proliferate and undergo differentiation [10]. Our *in vitro* cell culture data showed RA treatment directly promoted the proliferation of FAPs and maintained FAPs in a pre-differentiated state, which may further promote their proliferation. After peaking at about 4 days post-injury, the number of FAPs starts to decrease, which is caused by increased TNFα expression by pro-inflammatory cells [11]. Local inflammation induces TGFβ expression which prevents TNFα-induced apoptosis and induces the fibrotic differentiation of FAPs [11]. In other fibrosis models, differentiating myofibroblasts stimulated by TGFβ or other profibrotic signals acquire apoptosis resistance [49], [50], [51]. Our study showed that RA-supplementation maintained FAPs in an undifferentiated state and prevented TGFβ-induced differentiation, which might increase their sensitivity to TNFα-induced apoptosis. In addition, RA exhibits cell type-specific regulations on apoptosis, promoting apoptosis in certain cells especially cancer cells while preventing apoptosis in others [52], [53], [54], [55]. The proapoptotic regulation of RA is predominantly regulated by RAR and its transporter, CRABP-II [56]. Therefore, the role for RA on the proliferation of FAPs at the initial stage and their clearance at the later stage of muscle regeneration may be regulated by maintaining FAPs in an undifferentiated state.

Increased fat and fibrosis deposition were found in regenerated skeletal muscle of obese mice, hallmarks of incomplete muscle regeneration [22,24,25]. Using reporter mice, RA-signalling was found in large parts of the skeletal muscle tissue of mice and was activated after muscle injury [19]. A recent study also identified the differential expression of ALDH1As enzymes in specific cell populations in human skeletal muscle, and their expression was also linked to the disease of Duchenne muscular dystrophy [57]. Endogenous RA-signalling including RARγ and *Aldh1a2* are important for regeneration in skeletal and cardiac muscle after injury [19,21]. Attenuated RA-signalling in the skeletal muscle of obese mice was also found in this study and that might be responsible for the dysregulated proliferation, differentiation, and apoptosis of FAPs during regeneration. In our study, treatment of RA not only promotes the proliferation of FAPs but helps to clear FAPs timely after repair. Supplementation of RA to obese mice reduced fat degeneration and fibrotic tissue accumulation in regenerated skeletal muscle. Our data are consistent with therapeutic effects of RA on fibrotic diseases in the liver, lungs and kidneys [58].

In conclusion, RA-signalling maintains FAPs in an undifferentiated state, and promotes their proliferation at the early stage and apoptosis at the remodelling stage of muscle regeneration especially in obese mice. Supplementation of RA inhibits both adipogenic and fibrotic differentiation of FAPs, which are beneficial to muscle regeneration impaired due to obesity. Since excessive accumulation of intramuscular fat or fibrosis was also found in various muscular diseases such as dystrophies, denervation, diabetes, and ageing-related sarcopenia, RA-signalling mediators in FAPs are novel therapeutic targets to inhibit muscle loss and improve muscle functions. Due to the wide existence of RA-signalling in various cells, and fatty infiltration and fibrosis as key aetiological factors in the degeneration of tissues and organs, our discovery has wide clinical implications.

## Contributors

M. Du and L. Zhao designed and coordinated the whole study. L. Zhao, J.S. Son, B. Wang, Q. Tian, Y. Chen, and X. Liu conducted experimental works; L. Zhao and M. Du analysed the data, wrote, and edited the manuscript. L. Zhao, M. Du, J.M. de Avila and M.J. Zhu discussed and revised the manuscript. All authors read and approved the final version of the manuscript.

## Declarations of Competing Interests

The authors declare no conflict of interest.

## Appendix Supplementary materials

Image, application 1

Image, application 2
