# Supplementary material for: Retinoic acid signalling in fibro/adipogenic progenitors robustly enhances muscle regeneration
Source: eBioMedicine. 2020 Sep 24;60:103020. doi: 10.1016/j.ebiom.2020.103020 (PMC7519288; doi:10.1016/j.ebiom.2020.103020)
Supplement: Supplementary file 2 [file mmc2.docx]

**Supplementary material**

| **Table S1. Primer sequences used for real-time quantitative PCR** | | | | |
| --- | --- | --- | --- | --- |
| **Gene** | **Forward (5'-3')** | **Reverse (5'-3')** | **Size (bp)** | **Access No.** |
| *Actin* | TGTCCACCTTCCAGCAGATGT | AGCTCAGTAACAGTCCGCCTAG | 101 | NM_007393.5 |
| *Gapdh* | CACCATCTTCCAGGAGCGAG | CCTTCTCCATGGTGGTGAAGAC | 101 | NM_001289726.1 |
| *Pax7* | TTGGGGAACACTCCGCTGTGC | CAGGGCTTGGGAAGGGTTGGC | 115 | NM_011039.2 |
| *Myf5* | AAACTCCGGGAGCTCCGCCT | GGCAGCCGTCCGTCATGTCC | 125 | NM_008656.5 |
| *Myod* | TCTGGAGCCCTCCTGGCACC | CGGGAAGGGGGAGAGTGGGG | 100 | NM_010866.2 |
| *Myogenin* | GAGATCCTGCGCAGCGCCAT | CCCCGCCTCTGTAGCGGAGA | 97 | NM_031189.2 |
| *Igf1* | TCTACCTGGCGCTCTGCTTG | AATGCCTGTCTGAGGTGCCC | 176 | NM_031189.2 |
| *Il6* | GAGGATACCACTCCCAACAGACC | AAGTGCATCATCGTTGTTCATACA | 141 | NM_001314054.1 |
| *Wnt1* | TTCGGCAAGATCGTCAACCG | CTTGGCGCATCTCAGAGAAC | 313 | NM_021279.4 |
| *Wnt3*α | TGCGCTTCTGCAGGAACTAC | GGCATGGACAAAGGCTGACT | 185 | NM_009522.2 |
| *Wnt5*α | GTCCTTTGAGATGGGTGGTATC | ACCTCTGGGTTAGGGAGTGTCT | 175 | NM_001256224.2 |
| *Tcf4* | GGCGATGAGAACCTGCAAGA | GGTCCTCATCATCGTTATTGCTAGA | 115 | NM_013685.2 |
| *α-Sma* | ATGCTCCCAGGGCTGTTTTCCCAT | GTGGTGCCAGATCTTTTCCATGTCG | 191 | NM_007392.3 |
| *Col1*α | GCTCCTCTTAGGGGCCACT | CCACGTCTCACCATTGGGG | 103 | NM_007742.4 |
| *Col3*α | CTGTAACATGGAAACTGGGGAAA | CCATAGCTGAACTGAAAACCACC | 144 | NM_009930.2 |
| *Pref1* | AATAGACGTTCGGGCTTGCA | TCCAGGTCCACGCAAGTTCCATTGTT | 63 | NM_001190703.1 |
| *Sox9* | AGGTTTCAGATGCAGTGAGGAGCA | ACATACAGTCCAGGCAGACCCAAA | 81 | NM_011448.4 |
| *Klf2* | AAGAGCTCGCACCTAAAGGC | CTTTCGGTAGTGGCGGGTAA | 123 | NM_008452.2 |
| *Pdgfr*α *In* | AAAAGTGCCCATGCTCATTC | GCTTGGCAGAGCTACCTGTC | 98 | NM_001347718.1 |
| *Pdgfr*α *FL* | AGTGGCTACATCATCCCCCT | CCGAAGTCTGTGAGCTGTGT | 91 | NM_001083316.2 |
| *Rar*α | GAAAAAGAAGAAAGAGGCACCCAAGC | AGGTCAATGTCCAGGGAGACTCGTTG | 183 | NM_009024.2 |
| *Rar*β | AATGCTGGCTTCGGTCCTCTGACT | GCTTGCTGGGTCGTCGTTTTCTAATG | 217 | NM_011243.2 |
| *Rar*γ | ATGGATGACACCGAGACTGGGCTACT | CCTTTCTGCTCCCTTAGTGCTGATGC | 222 | NM_011244.4 |
| *Rxr*α | CGTGATAACAAAGACTGTACAGTGG | AGAATCTTCTCTACAGGCATGTCC | 270 | NM_011305.3 |
| *Rxr*β | CGTGATAACAAAGACTGTACAGTGG | GATGTTAGTCACTGGGTCATTTGG | 294 | NM_001205214.1 |
| *Rxr*γ | AAAGATCTCATCTACACCTGTCGG | GAGGGTGAAAAGTTGCTTATCTGC | 336 | NM_001159731.1 |
| *Aldh1*α*1* | GCGTGGTAAACATTGTCCCTGGTTA | GGGGTCAGAGGATTTCCAAGAACATA | 364 | NM_001361503.1 |
| *Aldh1α2* | AATCCAGCCACAGGAGAGCAAGTG | CACGGTGTTACCACAGCACAATGC | 447 | NM_009022.4 |
| *Aldh1*α*3* | AAGAGCAGGTCTACGGGGAGTTTGTG | GCTTTGTCCAGGTTTTTGGTGAACAC | 385 | NM_053080.3 |
| *C-ebp*α | CAAGAACAGCAACGAGTACCG | GTCACTGGTCAACTCCAGCAC | 124 | NM_001287514.1 |
| *Pparγ* | AGCTCCAAGAATACCAAAGTGCGAT | AGGTTCTTCATGAGGCCTGTTGTAGA | 98 | XM 017321456.1 |
| *Fabp4* | CGACAGGAAGGTGAAGAGCATCATA | CATAAACTCTTGTGGAAGTCACGCCT | 158 | NM 024406.2 |
| *Crbp1* | ACAAGGATCAGAAGCCACGGAACA | TCAGCTCCTATTTACCTGGGTCGG | 74 | NM_011254.5 |
| *Crabp1* | GCCAAGACGGGGATCAGTTCTACA | CGCCAAATGTCAGGATTAGCTCATC | 239 | NM_013496.3 |
| *Crabp2* | TCTAAAGAGAAAGCCACCTTGCTGC | CGTCATCTGCTGTCATTGTCAGGAT | 413 | NM_007759. |
| *Cyp26a1* | GCACAAGCAGCGAAAGAAGGTGATT | GGAAGAGAGAAGAGATTGCGGGTCA | 278 | NM_007811.2 |
| *Cyp26b1* | GGCAATCTTTTTCCTCTCTCTCTTCG | AACCAGTGACCAGTCTCTCCGATGAG | 385 | NM_001177713.1 |

**Fig. S1 Specific expression of the dominant negative retinoic acid receptor (*RARα403*) in fibro/adipogenic progenitors.** (**a**) A schematic showing the generation of *Pdgfrα-Cre ROSA^mT^/^mG^* double fluorescence mice. (**b**) In *Pdgfrα-cre ROSA^mT^/^mG^* mice, tdTomato (mT) fluorescence was widely expressed in the membrane of all cells prior to Cre-induced recombination. Tamoxifen injection (75mg/kg, three days) induced the expression of EGFP (mG) to replace the tdTomato fluorescence in *Pdgfrα*-cre expressing cells. Bars: 100μm. (**c**) Specific expression of *RARα403* transcript in FAPs of RARαDN mice. FAPs were isolated from the other cell populations (Non-FAPs) from the hind limb of WT and RARαDN mice using MACS as described in the Method Section. *RARα403* was amplified from cDNA and resolved by agarose gel. Expression of *Gapdh* was used as a control. (**d**) Relative expression of RA downstream-signalling mediators including *Crbp1*, *Cyp26a1*, *Rarα*, *Rarβ*, and *Rarγ*. Results represent the means ± SEM of three biological replicates. Statistics were analysed using an unpaired two-tailed Student’s t test (**d**). * p<0.05 shows significant difference between two groups.

**Fig. S2 Muscle regeneration with or without retinoic acid (RA) signalling in FAPs.** (**a**) Percentage of interstitial areas of regenerated muscle at 7 and 14 dpi. **(b)** The distribution of cross-sectional areas of regenerated myofibers (fibres with central nuclei) at 7 days post-injury (dpi). (**c**) Immunofluorescence staining of PAX7+ satellite cells in the *Tibialis anterior* muscle after RA supplementation but before cardiotoxin-induced injury. The total number of PAX7+ satellite cells from four randomly selected microscopic fields of each sample were counted and used for comparative analysis. Bars: 100μm. (**d**) Myogenic gene expression in injured muscle at 3 dpi. (**e**) Immunofluorescence analysis of PERILIPIN expression in regenerated muscle at 7 dpi. Bars: 200μm. (**f**) Immunofluorescence analysis of PDGFRα in skeletal muscle at 0, 3 and 7 dpi. Bars: 200μm. Results represent the means ± SEM of three mice per group at each time point. Statistics were analysed using a two-way ANOVA followed by Tukey’s multiple comparison. # shows significant interaction (p<0.05) between two factors while & and $ show significant difference (p<0.05) between genotypes (WT and RARαDN) and treatments (CON and RA), irrespectively. *p<0.05 and **p<0.01 show significant difference between two groups.

**Fig. S3 Retinoic acid (RA) rescues impaired muscle regeneration in obese mice through the regulation of FAPs.** (**a**) Relative mRNA expression of genes correlated with RA-signalling in normal diet (ND) and high fat diet (HFD) fed C57Bl6 mice. n=6. (**b**) Changes of body weights after ND and HFD feeding for 12 weeks. **(c)** The distribution of cross-sectional areas of regenerated myofibers (fibres with central nuclei) at 7 days post-injury (dpi). (**d**) Myogenic gene expression in injured muscle at 3 dpi. (**e**) Immunofluorescence analysis of PERILIPIN expression in regenerated muscle at 7 dpi. (**f**) Immunofluorescence analysis of PDGFRα+ FAPs in skeletal muscle at 0, 3 and 7 dpi. Bars: 200μm. Results represent the means ± SEM of three mice per group at each time point. Statistics were analysed using an unpaired two-tailed Student’s t test (a) or a one-way ANOVA (c, d). *<0.05 and **<0.01 show significant difference between two groups.

**Fig. S4 Full immunoblotting images of DESMIN and of β-ACTIN in the regenerated TA muscle at 14 dpi as listed in Fig. 1d.** The GFP shows the expression of DESMIN while the RFP shows the expression of β-ACTIN.


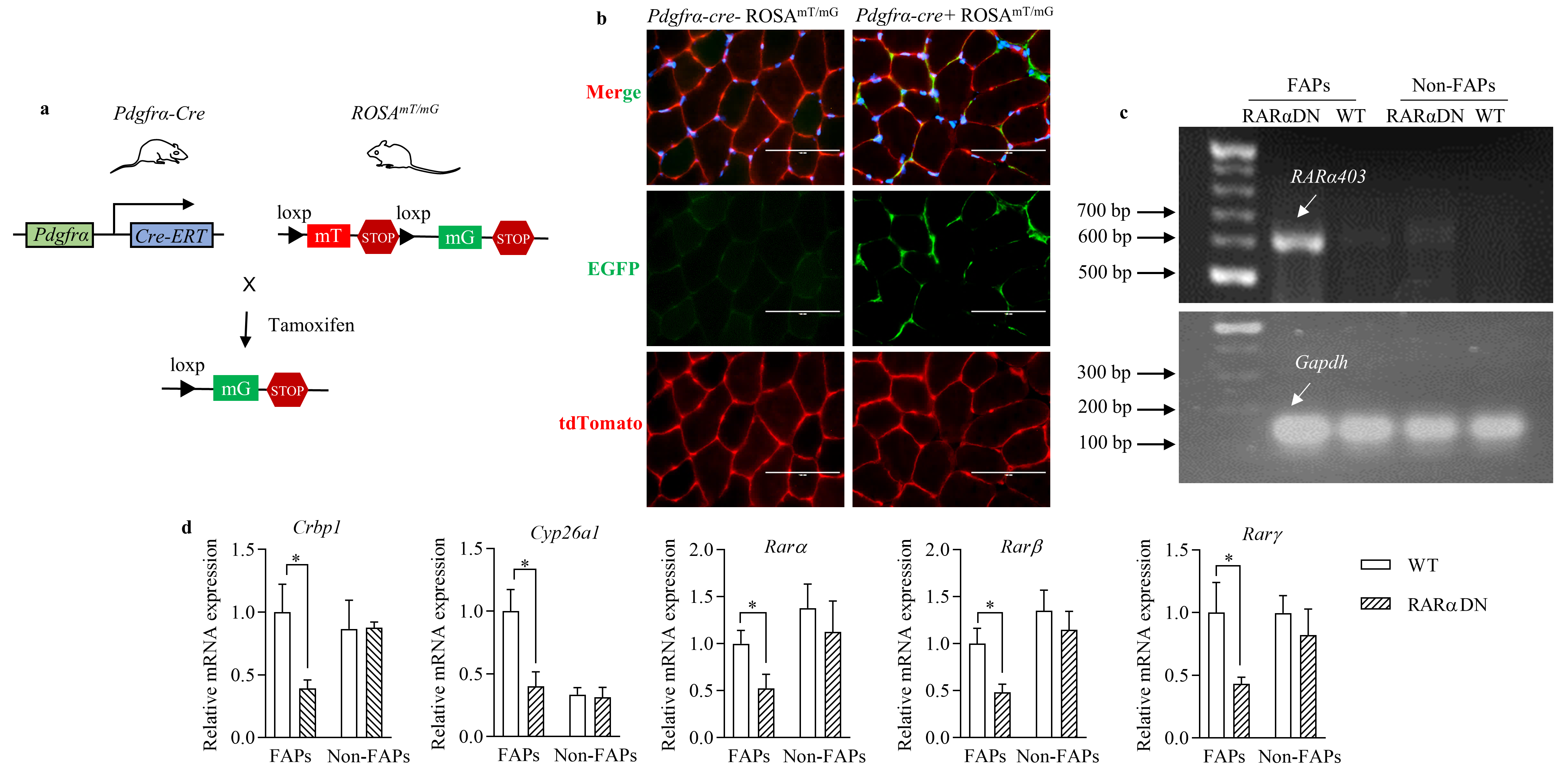


**Fig. S1**


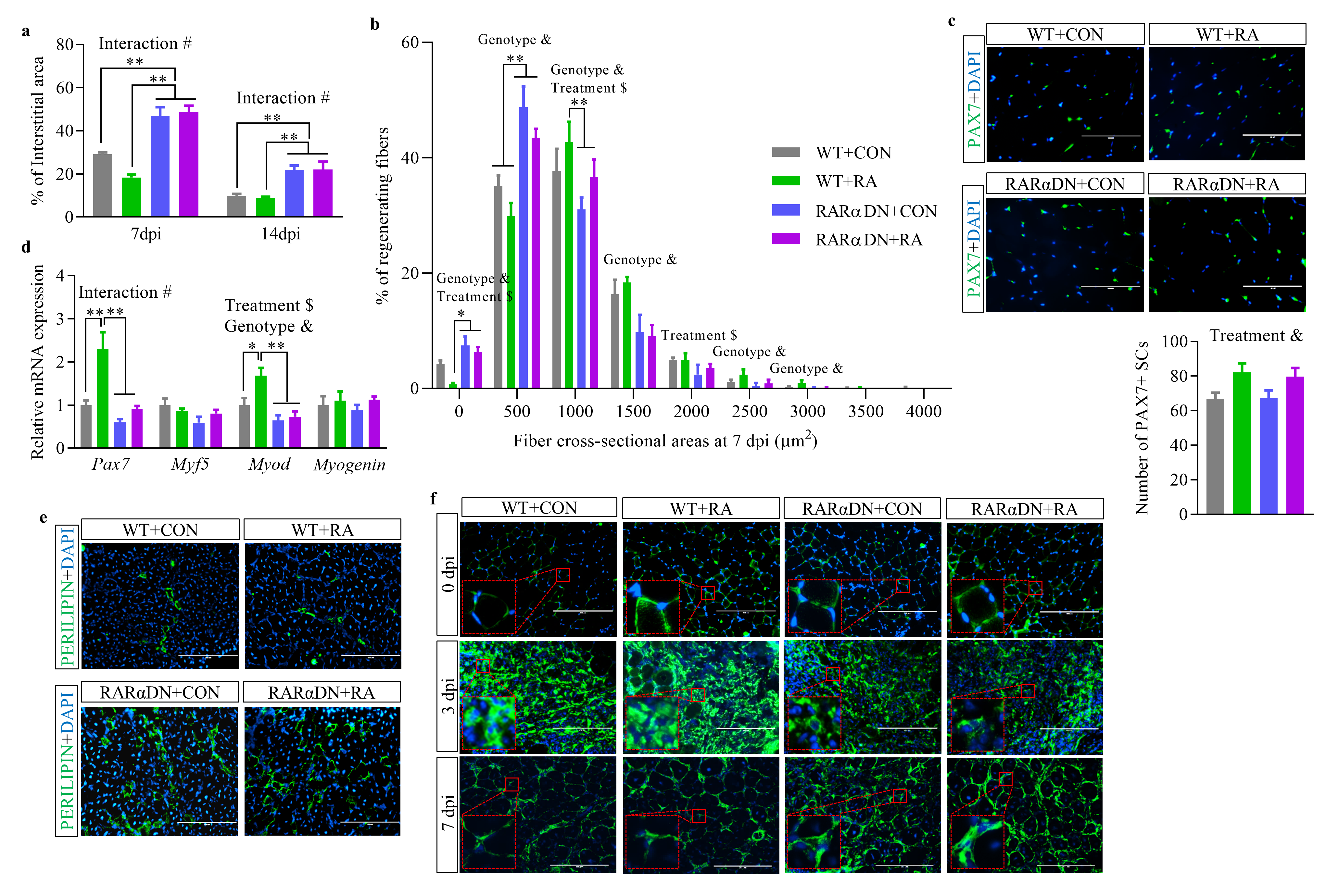


**Fig. S2**


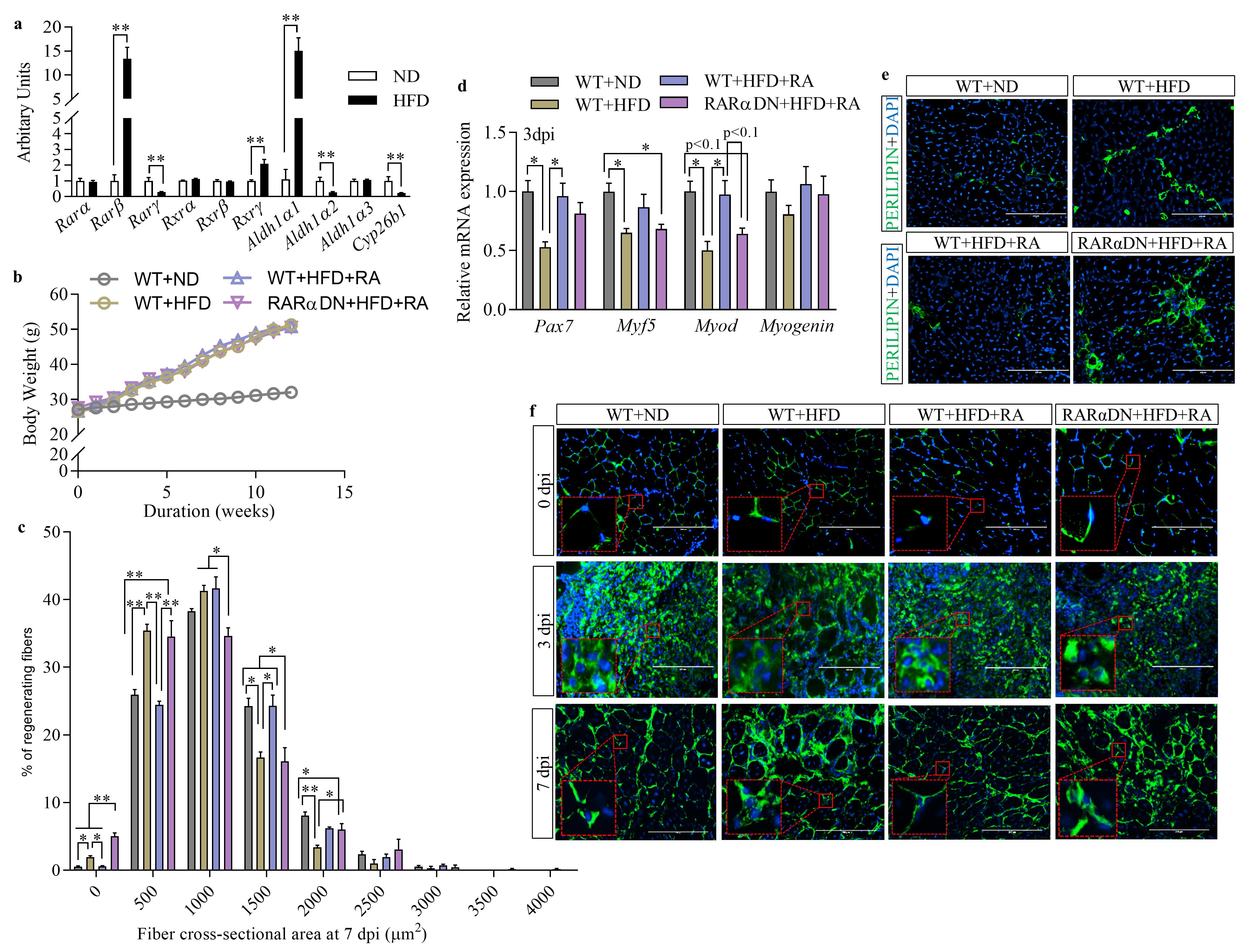


**Fig. S3**


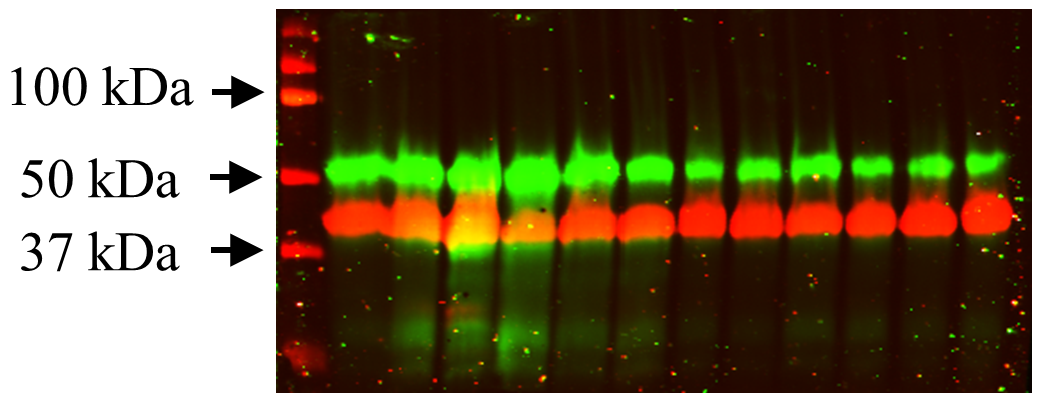


**Fig. S4**
